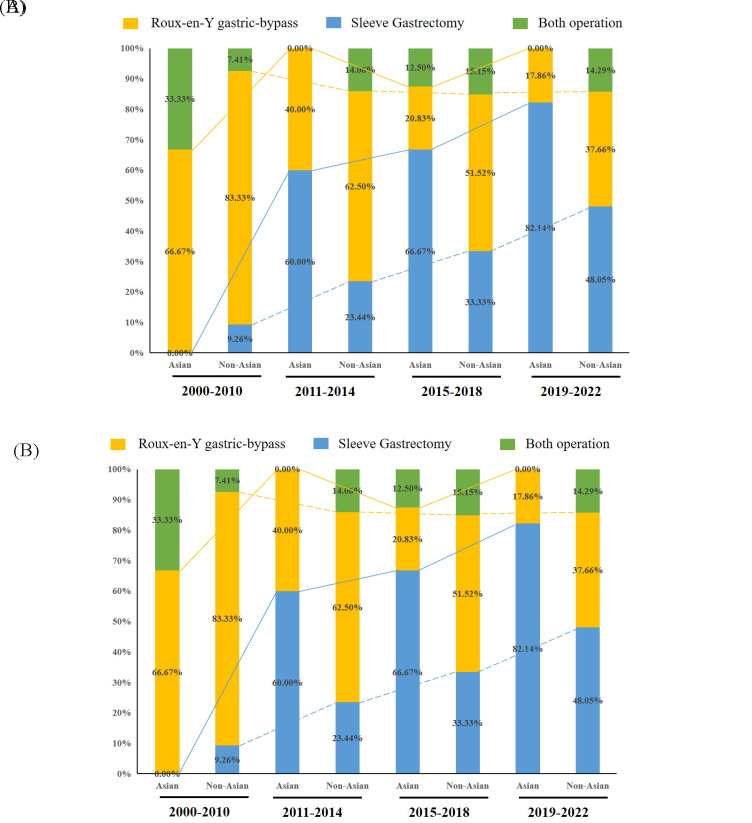# Characterization changes and research waste in randomized controlled trials of global bariatric surgery over the past 20 years: cross-sectional study

**DOI:** 10.1097/JS9.0000000000001013

**Published:** 2023-12-18

**Authors:** Ze-ning Huang, Wen-Wu Qiu, Qi-Chen He, Zhi-quan Zhang, Binbin Xu, Chang-yue Zheng, Chao-Hui Zheng, Jian Wei Xie, Jia-Bin Wang, Jian-Xian Lin, Qi-yue Chen, Long-Long Cao, Chang-ming Huang, Jun Lu, Ping Li

**Affiliations:** a Department of Gastric Surgery; b Department of General Surgery, Fujian Medical University Union Hospital; c Key Laboratory of Ministry of Education of Gastrointestinal Cancer, Fujian Medical University, Fuzhou, People’s Republic of China

**Keywords:** bariatric surgery, randomized controlled trials (RCTs), research waste

## Abstract

**Background:**

The results of several large randomized controlled trials (RCTs) have changed the clinical practice of bariatric surgery. However, the characteristics of global RCTs of bariatric surgery have not been reported internationally and whether there was research waste in these RCTs is unknown.

**Methods:**

Search ClinicalTrials.gov for bariatric surgery RCTs registered between January 2000 and December 2022 with the keywords ‘Roux-en-Y gastric-bypass’ and ‘Sleeve Gastrectomy’. The above analysis was conducted in January 2023.

**Results:**

A total of 326 RCTs were included in this study. The number of RCTs registered for sleeve gastrectomy and gastric bypass surgery increased year by year globally. Europe has always accounted for the largest proportion, Asia has gradually increased, and North America has decreased. A total of 171 RCTs were included in the analysis of waste, of which 74 (43.8%) were published. Of the 74 published RCTs, 37 (37/74, 50.0%) were judged to be adequately reported and 36 (36/74, 48.6%) were judged to have avoidable design defects. In the end, 143 RCTs (143/171, 83.6%) had at least one research waste. Body weight change as the primary endpoint (OR: 0.266, 95% CI: 0.103–0.687, *P*=0.006) and enrolment greater than 100 (OR: 0.349, 95% CI: 0.146–0.832, *P*=0.018) were independent protective factors for research waste.

**Conclusions:**

This study for the first time describes the characteristic changes of the mainstream RCT of bariatric surgery globally in the last 20 years and identifies a high research waste burden and predictive factor in this area, which provides reference evidence for carrying out bariatric surgery RCTs more rationally.

## Introduction

Highlights

- This study describes global changes in mainstream bariatric surgery (Roux-en-Y gastric-bypass and Sleeve Gastrectomy) over the last 20 years and identifies a high burden of research waste for the first time.
- This study finds registration and research waste distribution of two mainstream bariatric surgery randomised controlled trials.
- This study finds that research waste is an independent protective factor for guideline citations and prospective data reuse, which provides reference evidence for conducting bariatric surgery randomised controlled trials more rationally.

Bariatric surgery is the most effective treatment for patients with morbid obesity, and gastric bypass surgery has been regarded as the standard bariatric surgery in recent years^1^. In contrast, sleeve gastrectomy is a more convenient operation, with shorter operation times, and may be safer, so the use of sleeve gastrectomies is increasing^2^. To explore the differences between the two surgical procedures for the treatment of morbid obesity, including weight loss, changes in comorbidities, quality of life, and adverse events, an increasing number of randomised controlled trials (RCTs) have been carried out^3–6^. Although RCTs serve as the gold standard for new treatments and provide a high level of evidence-based medicine, research waste is a major challenge. This means that useless RCTs are conducted, limited resources are misused, and risks are increased in research participants. Chapman *et al*.^7^ showed that 85.2% of surgery-related RCTs contained research waste. Research waste can occur at any stage of the research cycle. First, research can be wasted because of avoidable design flaws, such as randomisation or poor blinding procedures, which reduce the credibility of the results. It is also possible to waste research by failing to consider previously conducted research. Second, it is a waste of time and money to conduct research that will never be published. The participants in such studies are at risk of unnecessary harm if the study is not published. Additionally, published research can be wasted owing to insufficient reporting, making it difficult or impossible to use and replicate research reports^8^. Ultimately, this waste prevents the results from being incorporated into diagnosis and treatment guidelines.

As the incidence of morbid obesity continues to increase, more RCTs in this area will be performed, and it is critical to minimise research waste so that new treatments can be translated into clinical practice appropriately, safely, and effectively. However, the research waste of bariatric surgery RCTs has not been elucidated. Therefore, this study aimed to explore the characteristics of RCTs associated with two major bariatric surgeries (Roux-en-Y gastric bypass and Sleeve Gastrectomy) conducted over the last 20 years and analyse the components of research waste in these RCTs to identify potential targets for improvement in the design of bariatric surgery RCTs. We also explored the impact of research waste on citations of published RCTs in treatment guidelines, as well as the reuse of prospective data.

## Methods

### Design and data

This cross-sectional study reviewed registered RCTs, and ethical issues such as blood samples, urine samples, salivary samples, and other secretions were not involved. The study process did not interfere with the treatment of patients and did not increase the risk to patients. It was exempt from research ethics approval and the requirement for informed consent by our hospital ethics committee. This study was conducted in line with the strengthening the reporting of cohort, cross-sectional and case–control studies in surgery (STROCSS) criteria^9^ (Supplemental Digital Content 1, http://links.lww.com/JS9/B599) (Supplemental Digital Content 2, http://links.lww.com/JS9/B600).

**Figure 1:**
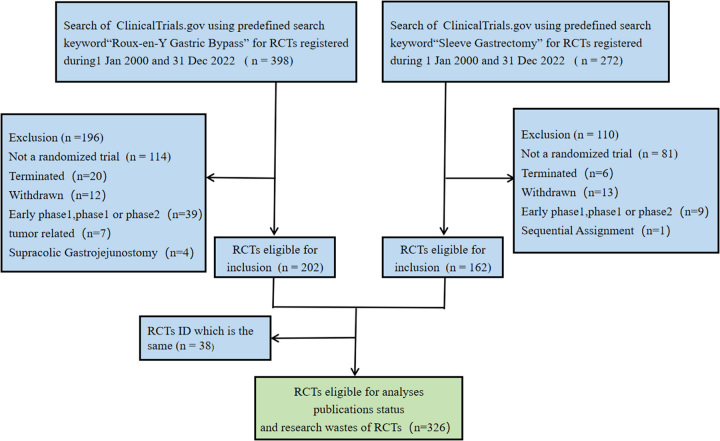
Flow chart.

Data for this study were obtained from ClinicalTrials.gov, a public registry of trials (https://clinicaltrials.gov/)^10^. We defined sleeve gastrectomy and gastric bypass as mainstream bariatric surgery. The ClinicalTrials.gov database was searched for the keyword ‘Roux-en-Y gastric-bypass’ or ‘Sleeve Gastrectomy’ on the same day (1 January 2023). Eligible trials were conducted between 1 January 2000 and 31 December 2022. The titles and abstracts of included studies were screened for eligibility. The exclusion criteria were: 1. nonrandomised trials; 2. early trials (Phase I or II clinical trials); 3. trials not related to bariatric surgery; and 4. trials terminated or withdrawn (Fig. 1). The assessment was conducted by two independent investigators (L.J. and W.-W.Q.), and discrepancies were resolved through consensus. The basic characteristics of the RCTs, such as study interventions, funding sources, and other key attributes, were audited by the ClinicalTrials.gov review staff^11^. Based on the institutional affiliations of the studies’ first authors, studies were grouped according to nationality. According to the World Bank Income classification, nationalities were further classified by income levels^12,13^. No RCTs have been conducted in low-income countries. Since there are few studies on low-income countries and middle-income countries (LMICs) (*n*=30), they were combined with high-income countries and middle-income countries (*n*=52), collectively referred to as middle-income countries (LMICs) (*n*=82) and compared with trials from high-income countries (HICs) (*n*=244). The RCTs were divided into Asian and non-Asian groups according to the affiliation of the principal investigator (PI) because Asian and non-Asian BMI classification criteria differ^14^.

### Study endpoints

RCT characteristics over the past 20 years and research waste were the primary outcomes of the study (i.e. nonpublication, inadequate reporting, and avoidable design flaws) to explore potentially modifiable research waste. Furthermore, we explored whether the published RCTs were referenced in guidelines and whether there was reuse of the prospective data because published RCTs are the precondition for the assessment of report adequacy and design flaws. All RCTs completed after December 2018 without publication were excluded from the analyses.

### Publication status

PubMed and Scopus were searched for the ClinicalTrials.gov identifier [National Clinical Trial (NCT) number], the name of the PI, and several keywords related to the RCTs. The manuscript information, including interventions, study participants, and dates of recruitment, was then reviewed to confirm the publication status. To further determine the publication status, our team contacted the corresponding PI if no corresponding manuscripts were available in PubMed or Scopus. In the absence of a response, the RCT was deemed unpublished by default. An article in a peer-reviewed journal with the full-text (print or online) was considered published^7^. The search was conducted by two independent investigators (W.-W.Q. and Q.-C.H.). The final search was conducted on 1 January 2023.

### Reporting adequacy assessment

The Consolidated Standards of Reporting Trials (CONSORT) guidelines were used to assess the adequacy of reporting for each manuscript. Pharmacological interventions accounted for 37 items, and nonpharmacological interventions accounted for 40 items. Each item was scored 1 point for reporting adequacy^15,16^. Therefore, the RCTs were categorised into pharmacological and nonpharmacological interventions according to the CONSORT statement. All manuscripts and supplementary correspondence materials were downloaded by a single investigator (Z.-Q.Z.). Secondly, Acrobat Pro PDF software version XI Pro (Adobe) was used by the investigator to remove author and journal information and to reduce biases. Two independent investigators (B.-B.X. and Z.-N.H.) reviewed and scored all manuscripts independently according to the CONSORT 2010 checklist. Every third manuscript was reviewed, and discrepancies were discussed and resolved by consensus. As the CONSORT reporting guideline statement contains many items, each assigned a point, the inter-rater agreement was not recorded. The full-text manuscript and supplementary correspondence materials were reviewed to determine whether a protocol was available^7^. As described previously, manuscripts that met 75% of the required items (28/37 pharmacological items and 30/40 nonpharmacological items) were considered adequately reported^7^.

### Design flaws

Two independent investigators (J.-W.X. and J.-B.W.) reviewed the blinded manuscripts to identify biases, such as selection bias, performance bias, detection bias, attrition bias, reporting bias, and others, using the Cochrane tool^17^. Items were classified as low risk, unclear, or high-risk. Every third manuscript was reviewed, and discrepancies were discussed and resolved by consensus. In the statistical analyses, items with an unclear bias risk were considered high-risk because a lack of clarity in key method descriptions influences judgments of an RCT’s usefulness for practical application. The presence of a relevant systematic review or justification for why one was not required in novel settings was also evaluated. For the RCT to be considered applicable, this had to be cited in the full-text manuscript and be considered capable of informing the necessity for carrying out the study. It was considered an avoidable design flaw if an article presented 1 of the previously listed biases or lacked a relevant systematic review citation.

### Referencing in guidelines and reuse of prospective data

Two independent researchers (J.L. and W.-W.Q.) searched Google Scholar databases for articles that cited each published RCT (http://www.scholar.google.com/)^18^. Secondly, each RCT article was manually reviewed according to the treatment or practice guidelines. In addition, we examined whether prospective data were reused for post-hoc analyses (e.g. whether data from the RCT were analysed for outcomes other than the preset primary or secondary outcomes)^19–21^. We assumed that the post-hoc analysis always cited the original RCT.

### Statistical analyses

Categorical data are presented as frequencies and percentages, while continuous data are expressed as medians and interquartile ranges. The Mann–Whitney *U* test was used to compare categorical and continuous data between groups. The χ^2^ test was used to compare categorical variables between groups, and Fisher’s test was performed if the number of samples was less than 5^22^. In the multiple regression analysis, a second-order polynomial equation was fitted to the data. To determine independent risk factors associated with research waste, simple and multivariate logistic regression models were used. Multivariate analysis was performed on variables with *P*<0.05. A two-step multivariate regression analysis was conducted, with the first step including all significant variables identified in the univariate analysis and the second step including all significant variables except Enrolment Time^23^. All statistical analyses were performed using SPSS statistical software version 26.0 and R statistical software version 4.0.6. Statistical significance was determined by *P*-values <0.05, and all tests were two-sided.

## Results

### Characteristics of RCTs over the 20 years

Between January 2000 and December 2022, 670 RCTs that met the inclusion criteria were retrieved. According to the inclusion and exclusion criteria, excluding gastric bypass surgery (*n*=196) and sleeve gastrectomy (*n*=110), 38 of the remaining 364 RCTs had the same NCT numbers, and 326 were included in the analysis (Fig. 1). Most RCTs were nonpharmacologically conducted (74.2%), and most PIs (72.9%) were from non-Asian countries, with 148 (45.4%) from Europe and 79 (24.2%) from North America. Most RCTs (244, 74.9%) were from high-income areas (HICs). A total of 262 (80.4%) clinical trials were registered in a single-centre. Only 40 (12.3%) RCTs received funding from external sources or manufacturers (Table 1).

**Table 1:** Characteristics of all RCTs.

	*N*=326
Enrolment time (months)	35.50±33.39
Registration time	
2000–2005	6 (1.8%)
2006–2010	51 (15.6%)
2011–2014	74 (22.6%)
2015–2018	90 (27.6%)
2019–2022	105 (32.2%)
No. of centres	
Monocentric	262 (80.4%)
Multicenter	64 (19.6%)
Primary outcome measures	
Nonweight loss	237 (72.6%)
Weight loss	89 (27.3%)
Primary outcome measures	
Nonanesthesia	262 (80.4%)
Anaesthesia	64 (19.6%)
Primary outcome measures	
Noncomorbility	255 (78.2%)
Comorbility	71 (21.8%)
Intervention	
Nonpharmacological	242 (74.2%)
Pharmacological	84 (25.7%)
Primary purpose	
Treatment	223 (68.4%)
Prevent	41 (12.6%)
Support care	18 (5.5%)
Other	44 (13.5%)
Study design	
Parallel group	291 (89.3%)
Crossover	31 (9.5%)
Factorial	4 (1.2%)
No. of arms	
2	272 (83.4%)
3	39 (12.0%)
≥4	15 (4.6%)
Blinding	
None/open label	140 (43.0%)
Single	84 (25.8%)
Double	46 (14.1%)
Triple	33 (10.1%)
Quardral	23 (7.0%)
Economic of PI region	
LMIC	82 (25.1%)
HIC	244 (74.9%)
Recruitment region	
Non-Asian	261 (80.0%)
Asian	65 (20.0%)
Recruitment region	
Africa	23 (7.0%)
Asian	65 (20.0%)
European	148 (45.4%)
North America	79 (24.2%)
South America	7 (2.2%)
Oceanian	4 (1.2%)
Operation	
Sleeve gastrectomy	124 (38.0%)
Roux-en-Y-bypass	164 (50.3%)
Both	38 (11.7%)
Funding type	
None/departmental	286 (87.7%)
Industry/other	40 (12.3%)
No. of participants	
≤100	208 (63.8%)
＞100	118 (36.1%)
No. of participants	
≤200	280 (85.8%)
＞200	46 (14.1%)

Over the past 20 years, the number of registered RCTs related to mainstream bariatric surgery has gradually increased, and the number of registered RCTs regarding sleeve gastrectomies tended to exceed that regarding gastric bypass surgery after 2016 (Figure S1, Supplemental Digital Content 3, http://links.lww.com/JS9/B601). The proportion of mainstream bariatric surgeries registered in Asia has increased, accounting for only 5.3% of RCTs in 2000–2010 and 26.7% in 2019–2022. The proportion of registered mainstream bariatric surgery RCTs decreased in North America, accounting for 43.9% of RCTs in 2000–2010 and 18.7% in 2019–2022, with Europe consistently accounting for the highest proportion at all stages (Figure S2, Supplemental Digital Content 4, http://links.lww.com/JS9/B602).

### Publication status

In this section, we exclude RCTs (*n*=155) whose end date was after December 2018 and that have not yet been published. Of the remaining 171 RCTs, 74 (43.3%) were published in peer-reviewed journals and were available for full-text review. Of these, 18 (24.5%) included pharmacological interventions and 56 (75.6%) included nonpharmacological interventions. Compared with published and unpublished RCTs, there were fewer in Europe (17.6%) than in North America (54.1%) (*P*=0.027), a higher proportion of Roux-en-Y-bypass RCTs (67.0 in Europe vs. 52.7% in North America), and 5.2 versus 18.9% involving both procedures in Europe and North America, respectively (*P*=0.014). In addition, compared with unpublished RCTs, enrolments were longer [odds ratio (OR): 1.017; 95% CI: 1.003–1.032; *P*=0.021) and registration was later in 2010 (OR: 4.988; 95% CI: 2.048–12.149; *P*<0.001), multicenter studies (OR: 2.932; 95% CI: 1.030–8.344; *P*=0.044), RCTs with greater than 100 participants (OR: 3.836; 95% CI: 1.792–8.215; *P*=0.001) was an independent risk factor for RCTS published (Table S1, Supplemental Digital Content 5, http://links.lww.com/JS9/B603, Table S2, Supplemental Digital Content 5, http://links.lww.com/JS9/B603).

### Adequacy of reporting

Table S3 (Supplemental Digital Content 5, http://links.lww.com/JS9/B603) shows the scores obtained for each of the 74 published RCTs in the CONSORT list. In pharmaceutical intervention-related RCTs (*n*=18), the reported deficiencies of concern included implementation of random sequence assignment (83.3%), study protocol (61.7%), mechanism of random sequence generation (50.0%), and absolute and relative errors of double-blind variables (50.0%). Among the RCTS with nonpharmaceutical interventions (*n*=56), the reported deficiencies were mainly in protocol compliance (57.2%), type and detail of randomisation (50.0%), and implementation of postintervention blinding (48.3%). Overall, 37 items (50.0%) were judged adequately reported. Well-reported RCTs were more likely to have a primary goal of treatment (83.8 vs. 56.8%, *P*=0.001) and a parallel design (94.6 vs. 78.4%, *P*=0.03) than under-reported RCTs (Table S4, Supplemental Digital Content 5, http://links.lww.com/JS9/B603).

### Design flaws

**Figure 2:**
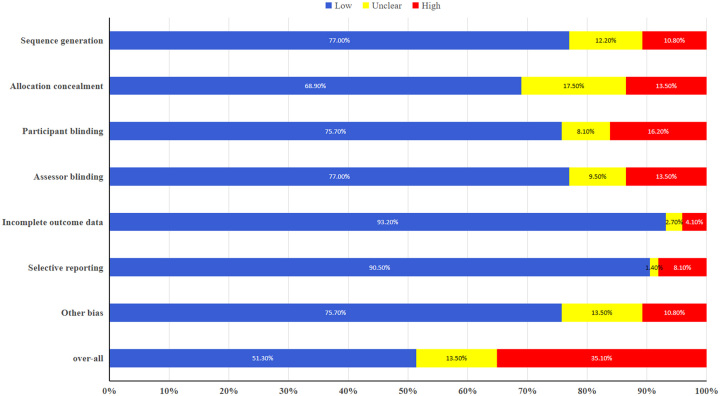
Risk-of-bias assessment.

Of the 74 published RCTs, 20 (27.0%) lacked a systematic literature review citing relevant RCTs. In addition, 36 RCTs (48.6%) had at least one feature, indicating a high or unclear risk of bias. Figure 2 shows that the most common causes of bias risk were random sequence assignment hiding (31.1%), the blinding practice of participants (24.3%), and other types of blinding practices (24.3%). When both factors were considered, 36 RCTs (48.6%) had avoidable design flaws. These RCTs were more likely to be treatment-type studies (84.2 vs. 55.6%, *P*=0.008) or have parallel designs (94.4 vs. 78.4%, *P*=0.025) (Table S5, Supplemental Digital Content 5, http://links.lww.com/JS9/B603).

### Research waste

**Figure 3:**
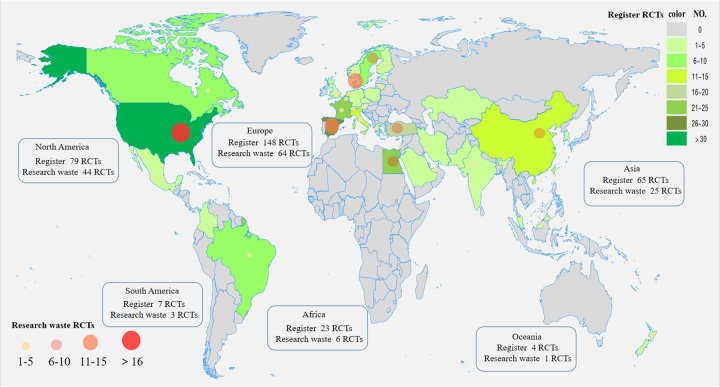
Registration and research waste distribution of two mainstream bariatric surgery randomized controlled trials during the past 20 years.

**Figure 4:**
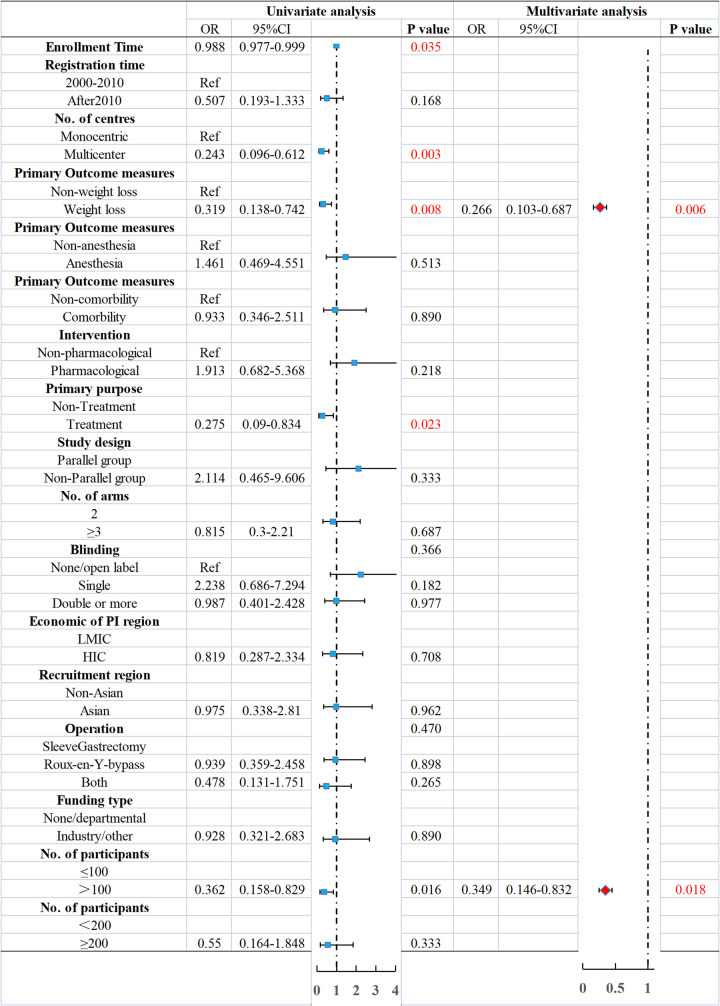
Forest map of the effect of key study characteristics on presence of research waste.

When combining publication status, adequate reporting, and avoidable design defects, 143 of the 171 RCTs (83.6%) contained at least one type of research waste. North America had a higher proportion of RCT study waste (30.8 vs. 14.3%, *P*=0.026). These RCTs were more likely to have a time of entry (33.2±29.0 vs. 47.6±41.3, *P*=0.027), a single-centre design (88.1 vs. 64.3%, *P*=0.002), a change in body weight as the primary endpoint (21.7 vs. 46.4%, *P*=0.006), the sample size was small (73.4 vs. 50.0%, *P*=0.014) (Table 2, Fig. 3). Multivariate analysis showed that studies with body weight change as the primary endpoint (OR: 0.266; 95% CI: 0.103–0.687; *P*=0.006), enrolment of greater than or equal to 100 participants (OR: 0.362; 95% CI: 0.158–0.829; *P*=0.018) was an independent protective factor for waste (Fig. 4).

**Table 2:** Characteristics of RCTs according to the presence of research waste.

	Absence of research waste (*n*=28)	Presence of research waste (*n*=143)	SMD	*P*
Enrolment time	47.6±41.3	33.2±29.0	0.4	0.027
Registration time			0.3	0.163
2000–2010	6 (21.4%)	50 (35.0%)		
After 2010	22 (78.6%)	93 (65.0%)		
No. of centres			0.6	0.002
Monocentric	18 (64.3%)	126 (88.1%)		
Multicenter	10 (35.7%)	17 (11.9%)		
Primary outcome measures			0.5	0.006
Nonweight loss	15 (53.6%)	112 (78.3%)		
Weight loss	13 (46.4%)	31 (21.7%)		
Primary outcome measures			0.1	0.511
Nonanesthesia	24 (85.7%)	115 (80.4%)		
Anaesthesia	4 (14.3%)	28 (19.6%)		
Primary outcome measures			0	0.89
Noncomorbility	22 (78.6%)	114 (79.7%)		
Comorbility	6 (21.4%)	29 (20.3%)		
Intervention			0.3	0.212
Nonpharmacological	23 (82.1%)	101 (70.6%)		
Pharmacological	5 (17.9%)	42 (29.4%)		
Primary purpose			0.9	0.006
Treatment	24 (85.7%)	89 (62.2%)		
Prevent	1 (3.6%)	21 (14.7%)		
Support care	3 (10.7%)	5 (3.5%)		
Other	0 (0.0%)	28 (19.6%)		
Study design			0.4	0.291
Parallel group	26 (92.9%)	123 (86.0%)		
Crossover	1 (3.6%)	18 (12.6%)		
Factorial	1 (3.6%)	2 (1.4%)		
No. of arms			0.1	0.687
2	22 (78.6%)	117 (81.8%)		
≥3	6 (21.4%)	26 (18.2%)		
Blinding			0.3	0.35
None/open label	14 (50.0%)	61 (42.7%)		
Single	4 (14.3%)	39 (27.3%)		
Double or more	10 (35.7%)	43 (30.1%)		
Economic of PI region			0.1	0.708
LMIC	5 (17.9%)	30 (21.0%)		
HIC	23 (82.1%)	113 (79.0%)		
Recruitment region			0	0.962
Non-Asian	23 (82.1%)	118 (82.5%)		
Asian	5 (17.9%)	25 (17.5%)		
Recruitment region			0.6	0.026
Africa	1 (3.6%)	6 (4.2%)		
Asian	5 (17.9%)	25 (17.5%)		
European	14 (50.0%)	64 (44.8%)		
North America	4 (14.3%)	44 (30.8%)		
South America	1 (3.6%)	3 (2.1%)		
Oceanian	3 (10.7%)	1 (0.7%)		
Operation			0.2	0.459
Sleeve gastrectomy	7 (25.0%)	41 (28.7%)		
Roux-en-Y-bypass	16 (57.1%)	88 (61.5%)		
Both	5 (17.9%)	14 (9.8%)		
Funding type			0	0.89
None/departmental	23 (82.1%)	119 (83.2%)		
Industry/other	5 (17.9%)	24 (16.8%)		
No. of participants			0.5	0.014
≤100	14 (50.0%)	105 (73.4%)		
＞100	14 (50.0%)	38 (26.6%)		
No. of participants			0.2	0.327
≤200	24 (85.7%)	131 (91.6%)		
＞200	4 (14.3%)	12 (8.4%)		
Impact Factor			0.6	0.017
＜10	16 (57.1%)	38 (82.6%)		
≥10	12 (42.9%)	8 (17.4%)		

Of the 74 published RCTs, 26 (45.7%) were cited in the guidelines. Research waste (OR: 0.124; 95% CI: 0.035–0.446; *P*=0.001) was an independent protective factor cited in the guidelines after the publication of RCTs (Tables S6, Supplemental Digital Content 5, http://links.lww.com/JS9/B603 and S7, Supplemental Digital Content 5, http://links.lww.com/JS9/B603). In addition, only 19 RCTs (25.7%) reused their prospective data and research waste (OR, 0.286; 95% CI: 0.090–0.903; *P*=0.033) as independent protective factors for the repeated use of prospective data in logistic two-step regression analyses (Tables S8, Supplemental Digital Content 5, http://links.lww.com/JS9/B603 and S9, Supplemental Digital Content 6, http://links.lww.com/JS9/B604).

## Discussion

This study is the first to analyse the characteristics of 326 large bariatric surgery-related RCTs over the past 20 years and found considerable research waste, with 83.6% of the RCTs having at least one feature of research waste. Of the 171 RCTs completed before December 2018, only 74 (43.2%) were published in peer-reviewed journals. In addition, only 26 RCTs (45.7%) were cited in the relevant guidelines, and 19 (25.7%) reused their prospective data. It has been found that RCTs with body weight change as the primary endpoint and enrolment of more than 100 participants generated less study waste, resulting in more guideline citations and more reuse of prospective data.

RCTs are the best way to minimise bias when evaluating novel treatments and should be conducted based on the severity of the disease burden^24^. However, the disease burden and research funding have been mismatched in previous studies^25^. According to a major research funder at the UK Department of Health and Social Care, it is important to conduct a systematic review of related research and existing evidence when deciding on further research^26^. It is not necessary to conduct new research unless the questions cannot be answered satisfactorily using existing evidence, and there has been an increase in RCTs related to mainstream bariatric surgery in the past 20 years; therefore, it is important to pay attention to the waste of research in such RCTs. In addition, this study found that the prevalence of sleeve gastrectomies increased over time, possibly due to the simple surgical procedure, fewer complications, and convenience of the procedure; therefore, sleeve gastrectomy is accepted by the majority of physicians and patients^27,28^. Suitable RCTs that avoids research waste is important for the success of sleeve gastrectomies.

Results reporting must be adequate for readers to judge whether the research applies to their practice^29^. Overall, most RCTs (50.0%) were identified as having a high reporting quality. Of particular concern; however, is the unsatisfactory quality of reporting on study randomisation and protocols in pharmacological research. Only ~38.3% of the trials provided their study protocols, which may have reduced the reader’s understanding of the overall design of the RCTs. The irrational use of randomisation also causes potential biases. This study also found inadequate protocol compliance and a lack of detailed descriptions of randomisation and blinding in nonpharmacological RCTs. In contrast to pharmacological research, surgical intervention has a high degree of variability in methods, techniques, and equipment, and the reliability and repeatability of research results directly depend on compliance with the research scheme and the degree of randomisation.

The effective size of the estimate may also be influenced by the design features of similar research conducted simultaneously when designing novel research. If the sample sizes of RCTs are insufficient, the statistical power may be affected, which is also part of the research waste. The research waste evaluation criteria used in this study included this part of the content. We stratified the sample size of RCTs according to less than or equal to 100 and less than or equal to 200 participants, and the results showed that enrolment of greater than or equal to 100 participants (OR: 0.362; 95% CI: 0.158–0.829; *P*=0.018) was an independent protective factor for waste after adjusting univariate analysis logistics regression. RCTs may suffer from allocation concealment, study blindness, and improper use of random allocation sequence methods, particularly if the endpoint is a subjective result^30^. Overall, our study found that 48.6% of the RCTs had avoidable design flaws. Random sequence allocation concealment flaws and evaluator blindness are common design flaws. In RCTs with design flaws, the study purpose and whether they were designed in parallel are likely to be closely related. Not all the limitations or design flaws in trials translate directly into research wastage. The relationship between trial design flaws and research waste is more complex than that assumed in this study and depends on the context. No RCT is perfect, and some limitations may need to be accepted.

There was an interesting finding in this study that the status of publications and research waste among bariatric surgery RCTs had nothing to do with the economic level of the PI’s region of origin, which indicated that low-and middle-income countries did not restrict the publication and cause research waste of RCTs. As long as a reasonable design scheme was adopted, high-quality RCTs could still be conducted^31^. However, publication and research waste were related to the geographical location of the PI, with a higher proportion of bariatric surgery RCTS published in Europe and a higher proportion of research waste published in North America. These findings may merely be statistical because many small RCTs in North America have investigated nonbody weight correlation as the primary endpoint in recent years. These attributes should not be considered equivalent to research waste. Scholars can preliminarily understand the latest information in related fields using these small RCTs, laying the groundwork for future large-scale RCTS in the future^32,33^.

Additionally, this study explored guideline citations and prospective data reuse. Correa *et al*. emphasised the advantages of prospective data and the importance of reuse. Not surprisingly, RCTs with earlier enrolment and less research waste were more likely to be cited and reuse prospective data. Clinical practice is more likely to benefit from RCTs published earlier because they have been investigated for a long time, and there is less research waste.

This study had the following limitations. First, quantifying research waste is challenging, which may not only be limited to the three factors defined in this study in different literature. Second, although ClinicalTrials.gov is a comprehensive clinical trial registry, several national RCTs were not included in this study. In addition, in this study, only Roux-en-Y gastric bypasses and sleeve gastrectomies were included, and other bariatric surgeries were not considered. Finally, some measurement errors may have occurred because some endpoints were collected by hand, and the use of additional detailed filters in the search could improve the reproducibility of the study. There are no further explanations or mechanisms for the correlation between small sample size, lack of external funding, and research waste in this study.

## Conclusion

This study describes global changes in mainstream bariatric surgery (Roux-en-Y gastric bypass and sleeve gastrectomy) over the last 20 years and identifies a high burden of research waste for the first time. Body weight change as the primary outcome (OR: 0.266; 95% CI: 0.103–0.687; *P*=0.006) and enrolment of greater than 100 participants (OR: 0.362; 95% CI: 0.158–0.829; *P*=0.018) were independent protective factors for research waste. In addition, our study found that research waste is an independent protective factor for guideline citations and prospective data reuse after the publication of RCTs for the first time. This provides reference evidence for conducting bariatric surgery RCTs more rationally and for reducing future research waste in this area.

## Ethical approval

Ethical approval was not required.

## Sources of funding

Fujian Research and TrainingGrants for Young and Middle-aged Leaders in Healthcare for Ping Li(No.[2022]954) Fujian Research andIraining Grants for Young and Middle -aged Leaders inHealthcare for Ju Lu (No.[ 2023 ] 26).Fujian third batch of "Innovation star" talent project (No.[2022]22).

## Author contribution

Z.-N.H., W.-W.Q., J.L., and P.L.: conception and design; Z.-N.H., W.-W.Q., P.L., and C.-M.H.: data analysis and interpretation. All authors contributed in manuscript writing, final approval of manuscript, collection and assembly of data, provision of study materials or patients, and accountable for all aspects of the work.

## Conflicts of interest disclosure

There are no conflicts of interest.

## Research registration unique identifying number (UIN)

1. Name of the registry: NA.
2. Unique identifying number or registration ID: NA.
3. Hyperlink to your specific registration (must be publicly accessible and will be checked): NA.

## Guarantor

Ze-Ning Huang, Wen-wu Qiu, and Ping Li.

## Data availability statement

The datasets generated during and/or analysed during the current study are available in the ClinicalTrials.gov, a public registry of trials (https://clinicaltrials.gov/).

## Provenance and peer review

Not commissioned, externally peer-reviewed.

## Supplementary Material

SUPPLEMENTARY MATERIAL

## Supplementary Material

**Figure:**
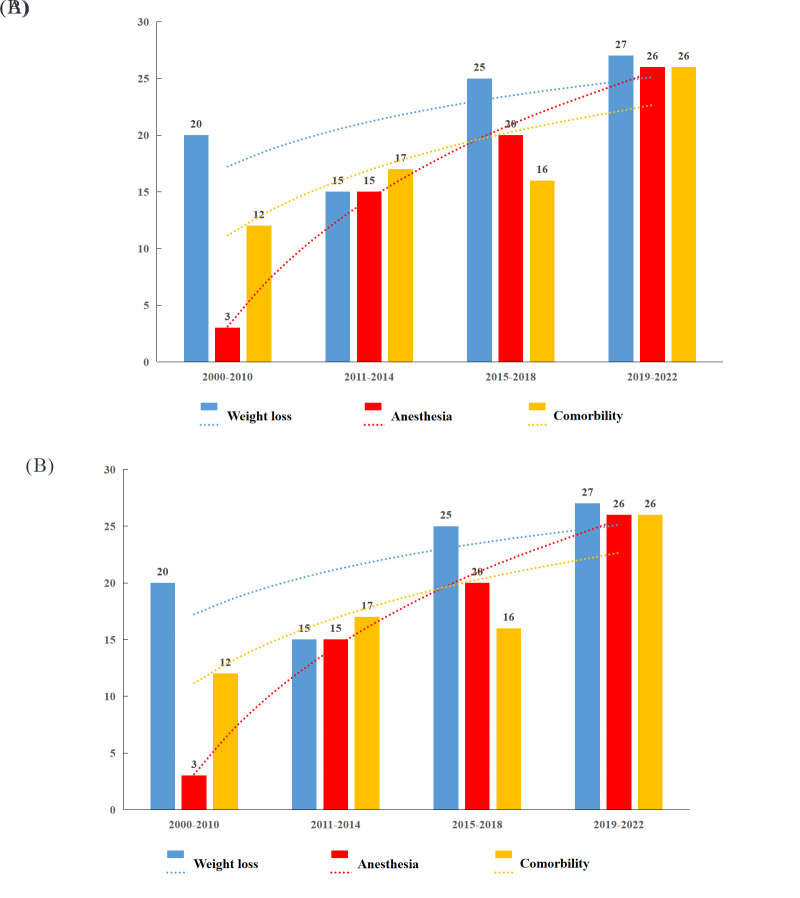


**Figure:**