# Supplementary material for: Characterization changes and research waste in randomized controlled trials of global bariatric surgery over the past 20 years: cross-sectional study
Source: Int J Surg. 2023 Dec 18;110(3):1420–9. doi: 10.1097/JS9.0000000000001013 (PMC10942146; doi:10.1097/JS9.0000000000001013)
Supplement: SUPPLEMENTARY MATERIAL [file js9-110-1420-s005.docx]

**Table S1. Characteristics of RCTs completed before 2018, December according to publication status.**

|  | **Not published (n=97)** | **Published (n=74)** | **SMD** | **P** |
| --- | --- | --- | --- | --- |
| **Enrollment Time** | 30.4 ± 22.0 | 42.3 ± 40.1 | 0.4 (0.1, 0.7) | 0.014 |
| **Registration time** |  |  | 0.4 (0.1, 0.7) | 0.007 |
| 2000-2010 | 40 (41.2%) | 16 (21.6%) |  |  |
| After2010 | 57 (58.8%) | 58 (78.4%) |  |  |
| **No. of centres** |  |  | 0.5 (0.2, 0.8) | 0.002 |
| Monocentric | 89 (91.8%) | 55 (74.3%) |  |  |
| Multicenter | 8 (8.2%) | 19 (25.7%) |  |  |
| **Primary Outcome measures** |  |  | 0.4 (0.1, 0.7) | 0.014 |
| Non-weight loss | 79 (81.4%) | 48 (64.9%) |  |  |
| Weight loss | 18 (18.6%) | 26 (35.1%) |  |  |
| **Primary Outcome measures** |  |  | 0.1 (-0.2, 0.4) | 0.465 |
| Non-anesthesia | 77 (79.4%) | 62 (83.8%) |  |  |
| Anesthesia | 20 (20.6%) | 12 (16.2%) |  |  |
| **Primary Outcome measures** |  |  | 0.2 (-0.1, 0.5) | 0.275 |
| Non-comorbility | 80 (82.5%) | 56 (75.7%) |  |  |
| Comorbility | 17 (17.5%) | 18 (24.3%) |  |  |
| **Intervention** |  |  | 0.1 (-0.2, 0.4) | 0.419 |
| Non-pharmacological | 68 (70.1%) | 56 (75.7%) |  |  |
| Pharmacological | 29 (29.9%) | 18 (24.3%) |  |  |
| **Primary purpose** |  |  | 0.2 (-0.1, 0.5) | 0.581 |
| Treatment | 61 (62.9%) | 52 (70.3%) |  |  |
| Prevent | 13 (13.4%) | 9 (12.2%) |  |  |
| Support care | 4 (4.1%) | 4 (5.4%) |  |  |
| Other | 19 (19.6%) | 9 (12.2%) |  |  |
| **Study design** |  |  | 0.1 (-0.2, 0.4) | 0.88 |
| Parallel group | 85 (87.6%) | 64 (86.5%) |  |  |
| Crossover | 10 (10.3%) | 9 (12.2%) |  |  |
| Factorial | 2 (2.1%) | 1 (1.4%) |  |  |
| **No. of arms** |  |  | 0.1 (-0.2, 0.4) | 0.648 |
| 2 | 80 (82.5%) | 59 (79.7%) |  |  |
| ≥3 | 17 (17.5%) | 15 (20.3%) |  |  |
| **Blinding** |  |  | 0.4 (0.1, 0.7) | 0.063 |
| None/open label | 35 (36.1%) | 40 (54.1%) |  |  |
| Single | 28 (28.9%) | 15 (20.3%) |  |  |
| Double or more | 34 (35.1%) | 19 (25.7%) |  |  |
| **Economic of PI region** |  |  | 0.1 (-0.2, 0.4) | 0.412 |
| LMIC | 22 (22.7%) | 13 (17.6%) |  |  |
| HIC | 75 (77.3%) | 61 (82.4%) |  |  |
| **Recruitment region** |  |  | 0.1 (-0.2, 0.4) | 0.69 |
| Non-Asian | 79 (81.4%) | 62 (83.8%) |  |  |
| Asian | 18 (18.6%) | 12 (16.2%) |  |  |
| **Recruitment region** |  |  | 0.6 (0.2, 0.9) | 0.027 |
| Africa | 4 (4.1%) | 3 (4.1%) |  |  |
| Asian | 18 (18.6%) | 12 (16.2%) |  |  |
| European | 38 (39.2%) | 40 (54.1%) |  |  |
| North America | 35 (36.1%) | 13 (17.6%) |  |  |
| South America | 2 (2.1%) | 2 (2.7%) |  |  |
| Oceanian | 0 (0.0%) | 4 (5.4%) |  |  |
| **Operation** |  |  | 0.4 (0.1, 0.8) | 0.014 |
| SleeveGastrectomy | 27 (27.8%) | 21 (28.4%) |  |  |
| Roux-en-Y-bypass | 65 (67.0%) | 39 (52.7%) |  |  |
| Both | 5 (5.2%) | 14 (18.9%) |  |  |
| **Funding type** |  |  | 0.1 (-0.2, 0.4) | 0.524 |
| None/departmental | 79 (81.4%) | 63 (85.1%) |  |  |
| Industry/other | 18 (18.6%) | 11 (14.9%) |  |  |
| **No. of participants** |  |  | 0.7 (0.4, 1.0) | <0.001 |
| ≤100 | 80 (82.5%) | 39 (52.7%) |  |  |
| ＞100 | 17 (17.5%) | 35 (47.3%) |  |  |
| **No. of participants** |  |  | 0.5 (0.2, 0.8) | 0.001 |
| ≤200 | 94 (96.9%) | 61 (82.4%) |  |  |
| ＞200 | 3 (3.1%) | 13 (17.6%) |  |  |

PI indicates principal investigator.

**Table S2.Adjusted logistic regression analysis of the effect of key study characteristics on Public status**

|  | **Univariate analysis** |  | **Multivariate analysis** |  |
| --- | --- | --- | --- | --- |
|  | **OR（95% CI）** | **P** | **OR（95% CI）** | **P** |
| **Enrollment Time** | 1.013(1.002-1.023) | 0.019 | 1.017(1.003-1.032) | 0.021 |
| **Registration time** |  |  |  |  |
| 2000-2010 |  |  |  |  |
| After2010 | 2.544(1.282-5.048) | 0.008 | 4.988(2.048-12.149) | ＜0.001 |
| **No. of centres** |  |  |  |  |
| Monocentric |  |  |  |  |
| Multicenter | 3.843(1.575-9.376) | 0.003 | 2.932(1.030-8.344) | 0.044 |
| **Primary Outcome measures** |  |  |  |  |
| Non-weight loss |  |  |  |  |
| Weight loss | 2.377(1.181-4.787) | 0.015 |  |  |
| **Primary Outcome measures** |  |  |  |  |
| Non-anesthesia |  |  |  |  |
| Anesthesia | 0.745(0.338-1.642) | 0.465 |  |  |
| **Primary Outcome measures** |  |  |  |  |
| Non-comorbility |  |  |  |  |
| Comorbility | 1.513(0.718-3.188) | 0.277 |  |  |
| **Intervention** |  |  |  |  |
| Non-pharmacological |  |  |  |  |
| Pharmacological | 0.783(0.307-2.001) | 0.61 |  |  |
| **Primary purpose** |  |  |  |  |
| Non-Treatment |  |  |  |  |
| Treatment | 1.395(0.731-2.663) | 0.313 |  |  |
| **Study design** |  |  |  |  |
| Parallel group |  |  |  |  |
| Non-Parallel group | 1.107(0.45-2.721) | 0.825 |  |  |
| **No. of arms** |  |  |  |  |
| 2 |  |  |  |  |
| ≥3 | 1.196(0.553-2.588) | 0.649 |  |  |
| **Blinding** |  |  |  |  |
| None/open label |  | 0.066 |  |  |
| Single | 0.469(0.216-1.016) | 0.055 |  |  |
| Double or more | 0.489(0.238-1.006) | 0.052 |  |  |
| **Economic of PI region** |  |  |  |  |
| LMIC |  |  |  |  |
| HIC | 1.376(0.641-2.956) | 0.413 |  |  |
| **Recruitment region** |  |  |  |  |
| Non-Asian |  |  |  |  |
| Asian | 0.849(0.381-1.895) | 0.69 |  |  |
| **Operation** |  |  |  |  |
| Sleeve Gastrectomy |  | 0.022 |  |  |
| Roux-en-Y-bypass | 0.771(0.385-1.545) | 0.464 |  |  |
| Both | 3.6(1.118-11.594) | 0.032 |  |  |
| **Funding type** |  |  |  |  |
| None/departmental |  |  |  |  |
| Industry/other | 0.766(0.338-1.74) | 0.525 |  |  |
| **No. of participants** |  |  |  |  |
| ≤100 |  |  |  |  |
| ＞100 | 4.223(2.109-8.457) | 0 | 3.836(1.792-8.215) | 0.001 |
| **No. of participants** |  |  |  |  |
| ＜200 |  |  |  |  |
| ≥200 | 6.678(1.827-24.406) | 0.004 |  |  |

OR indicates odd ratio

**Table S3. Compliance with items of the CONSORT 2010 Checklist**

| **CONSORT Item** | | **Pharmacological** | **NPI** |
| --- | --- | --- | --- |
|  |  | **n=18** | **n=56** |
| **1a** | Identification as a randomised trial in the title | 10 (55.5%) | 42 (75.0%) |
| **1b** | Structured summary of trial design, methods, results, and conclusions | 17 (94.4%) | 55 (98.2%) |
| **2a** | Scientific background and explanation of rationale | 18(100.0%) | 56 (100.0%) |
| **2b** | Specific objectives or hypotheses | 18 (100.0%) | 56(100.0%) |
| **3a** | Description of trial design (such as parallel, factorial) including allocation ratio | 13(72.2%) | 49 (87.5%) |
| **3b*** | Important changes to methods after trial commencement (such as eligibility criteria), with reasons | - | - |
| **4a** | Eligibility criteria for participants | 15 (83.3%) | 54 (96.4%) |
| **4b** | Settings and locations where the data were collected | 13 (72.2%) | 55 (98.2%) |
| **5** | The interventions for each group with sufficient details to allow replication | 17 (94.4%) | 55 (98.2%) |
| **5A**** | Description of the components of the interventions and, if applicable, the procedure for individualizing treatment | N/A | 55 (98.2%) |
| **5B**** | Details of how the interventions were standardized | N/A | 48 (85.7%) |
| **5C**** | Details of how the adherence of care provers with the protocol was assessed or enhanced | N/A | 24 (42.8%) |
| **6a** | Completely defined pre-specified primary and secondary outcome measures | 16 (88.8%) | 52(92.8%) |
| **6b*** | Any changes to trial outcomes after the trial commenced, with reasons | - | - |
| **7a** | How sample size was determined | 10 (55.5%) | 41 (73.2%) |
| **7b*** | When applicable, explanation of any interim analyses and stopping guidelines | - | - |
| **8a** | Method used to generate the random allocation sequence | 50 (78.1%) | 44 (78.5%) |
| **8b** | Type of randomisation; details of any restriction (such as blocking and block size) | 11 (61.1%) | 28 (50.0%) |
| **9** | Mechanism used to implement the random allocation sequence | 3 (16.6%) | 39 (69.6%) |
| **10** | Who generated the random allocation sequence, enrolled participants, and assigned participants to interventions | 9 (50.0%) | 39 (69.6%) |
| **11a** | If done, who was blinded after assignment to interventions and how | 1 1(61.1%) | 29(51.7%) |
| **11b*** | If relevant, description of the similarity of interventions | - | - |
| **12a** | Statistical methods used to compare groups for primary and secondary outcomes | 17(94.4%) | 55 (98.2%) |
| **12b*** | Methods for additional analyses, such as subgroup analyses and adjusted analyses | - | - |
| **13a** | The numbers of participants who were randomised, received treatment, and analysed for the primary outcome | 14 (77.7%) | 55 (98.2%) |
| **13b** | For each group, losses and exclusions after randomisation, together with reasons | 15 (83.3%) | 53 (94.6%) |
| **14a** | Dates defining the periods of recruitment and follow-up | 12 (66.6%) | 51 (91.0%) |
| **14b*** | Why the trial ended or was stopped | - | - |
| **15** | A table showing baseline demographic and clinical characteristics for each group | 15 (83.3%) | 52 (92.8%) |
| **16** | For each group, number of participants analysed and whether the analysis was by original assigned groups | 14 (77.7%) | 50 (89.2%) |
| **17a** | For each primary and secondary outcome, results for each group, and the estimated effect size and its precision | 15 (83.3%) | 51 (91.0%) |
| **17b** | For binary outcomes, presentation of both absolute and relative effect sizes is recommended | 9 (50.0%) | 35 (62.5%) |
| **18*** | Results of any other analyses performed, including subgroup analyses and adjusted analyses | - | - |
| **19** | All important harms or unintended effects in each group | 15(83.3%) | 45 (80.3%) |
| **20** | Trial limitations, addressing sources of potential bias, imprecision, and, if relevant, multiplicity of analyses | 14 (77.7%) | 43 (76.7%) |
| **21** | Generalisability (external validity, applicability) of the trial findings | 13 (72.2%) | 46 (82.1%) |
| **22** | Interpretation consistent with results, balancing benefits and harms, and considering other relevant evidence | 17(94.4%) | 49 (87.5%) |
| **23** | Registration number and name of trial registry | 23 (100.0%) | 56 (100.0%) |
| **24** | Where the full trial protocol can be accessed, if available | 7 (38.3%) | 34(60.7%) |
| **25** | Sources of funding and other support (such as supply of drugs), role of funders | 13 (72.2%) | 40 (71.4%) |
| *a conditional item for which not all manuscripts were scored  ** items relate to non-pharmacological (NPI) RCTs only | | | |

**Table S4. Characteristics of Randomized Clinical Trials according to Reporting Adequacy**

|  | **Inadequate reporting (n=37)** | **Adequate reporting （N=37）** | **SMD** | **P-value** |
| --- | --- | --- | --- | --- |
| **Enrollment Time** | 35.3 ± 37.9 | 49.4 ± 41.6 | 0.4 | 0.133 |
| **Registration time** |  |  | 0.1 | 0.572 |
| 2000-2010 | 7 (18.9%) | 9 (24.3%) |  |  |
| After2010 | 30 (81.1%) | 28 (75.7%) |  |  |
| **No. of centres** |  |  | 0.4 | 0.062 |
| Monocentric | 31 (83.8%) | 24 (64.9%) |  |  |
| Multicenter | 6 (16.2%) | 13 (35.1%) |  |  |
| **Primary Outcome measures** |  |  | 0.2 | 0.33 |
| Non-weight loss | 26 (70.3%) | 22 (59.5%) |  |  |
| Weight loss | 11 (29.7%) | 15 (40.5%) |  |  |
| **Primary Outcome measures** |  |  | 0.1 | 0.528 |
| Non-anesthesia | 30 (81.1%) | 32 (86.5%) |  |  |
| Anesthesia | 7 (18.9%) | 5 (13.5%) |  |  |
| **Primary Outcome measures** |  |  | 0 | 1 |
| Non-comorbility | 28 (75.7%) | 28 (75.7%) |  |  |
| Comorbility | 9 (24.3%) | 9 (24.3%) |  |  |
| **Intervention** |  |  | 0.3 | 0.278 |
| Non-pharmacological | 26 (70.3%) | 30 (81.1%) |  |  |
| Pharmacological | 11 (29.7%) | 7 (18.9%) |  |  |
| **Primary purpose** |  |  | 1.1 | <0.001 |
| Treatment | 21 (56.8%) | 31 (83.8%) |  |  |
| Prevent | 8 (21.6%) | 1 (2.7%) |  |  |
| Support care | 0 (0.0%) | 4 (10.8%) |  |  |
| Other | 8 (21.6%) | 1 (2.7%) |  |  |
| **Study design** |  |  | 0.6 | 0.03 |
| Parallel group | 29 (78.4%) | 35 (94.6%) |  |  |
| Crossover | 8 (21.6%) | 1 (2.7%) |  |  |
| Factorial | 0 (0.0%) | 1 (2.7%) |  |  |
| **No. of arms** |  |  | 0.1 | 0.772 |
| 2 | 29 (78.4%) | 30 (81.1%) |  |  |
| ≥3 | 8 (21.6%) | 7 (18.9%) |  |  |
| **Blinding** |  |  | 0.2 | 0.585 |
| None/open label | 20 (54.1%) | 20 (54.1%) |  |  |
| Single | 9 (24.3%) | 6 (16.2%) |  |  |
| Double or more | 8 (21.6%) | 11 (29.7%) |  |  |
| **Economic of PI region** |  |  | 0.1 | 0.76 |
| LMIC | 6 (16.2%) | 7 (18.9%) |  |  |
| HIC | 31 (83.8%) | 30 (81.1%) |  |  |
| **Recruitment region** |  |  | 0.1 | 0.528 |
| Non-Asian | 32 (86.5%) | 30 (81.1%) |  |  |
| Asian | 5 (13.5%) | 7 (18.9%) |  |  |
| **Recruitment region** |  |  | 0.5 | 0.529 |
| Africa | 2 (5.4%) | 1 (2.7%) |  |  |
| Asian | 5 (13.5%) | 7 (18.9%) |  |  |
| European | 22 (59.5%) | 18 (48.6%) |  |  |
| North America | 7 (18.9%) | 6 (16.2%) |  |  |
| South America | 0 (0.0%) | 2 (5.4%) |  |  |
| Oceanian | 1 (2.7%) | 3 (8.1%) |  |  |
| **Operation** |  |  | 0.5 | 0.166 |
| Sleeve Gastrectomy | 14 (37.8%) | 7 (18.9%) |  |  |
| Roux-en-Y-bypass | 16 (43.2%) | 23 (62.2%) |  |  |
| Both | 7 (18.9%) | 7 (18.9%) |  |  |
| **Funding type** |  |  | 0.2 | 0.327 |
| None/departmental | 33 (89.2%) | 30 (81.1%) |  |  |
| Industry/other | 4 (10.8%) | 7 (18.9%) |  |  |
| **No. of participants** |  |  | 0.1 | 0.816 |
| ≤100 | 20 (54.1%) | 19 (51.4%) |  |  |
| ＞100 | 17 (45.9%) | 18 (48.6%) |  |  |
| **No. of participants** |  |  | 0.1 | 0.76 |
| ≤200 | 31 (83.8%) | 30 (81.1%) |  |  |
| ＞200 | 6 (16.2%) | 7 (18.9%) |  |  |

**Table S5. Characteristics of Randomized Clinical Trials according to Presence of Avoidable Design Flaws**

|  | **Absence of design flaw (n=38)** | **Presence of design flaw （N=36）** | **SMD** | **P-value** |
| --- | --- | --- | --- | --- |
| **Enrollment Time** | 45.9 ± 41.3 | 38.5 ± 39.1 | 0.2 | 0.429 |
| **Enrollment Time** |  |  | 0.1 | 0.658 |
| 2000-2010 | 9 (23.7%) | 7 (19.4%) |  |  |
| After2010 | 29 (76.3%) | 29 (80.6%) |  |  |
| **No. of centres** |  |  | 0.3 | 0.232 |
| Monocentric | 26 (68.4%) | 29 (80.6%) |  |  |
| Multicenter | 12 (31.6%) | 7 (19.4%) |  |  |
| **Primary Outcome measures** |  |  | 0.4 | 0.075 |
| Non-weight loss | 21 (55.3%) | 27 (75.0%) |  |  |
| Weight loss | 17 (44.7%) | 9 (25.0%) |  |  |
| **Primary Outcome measures** |  |  | 0 | 0.919 |
| Non-anesthesia | 32 (84.2%) | 30 (83.3%) |  |  |
| Anesthesia | 6 (15.8%) | 6 (16.7%) |  |  |
| **Primary Outcome measures** |  |  | 0.2 | 0.5 |
| Non-comorbility | 30 (78.9%) | 26 (72.2%) |  |  |
| Comorbility | 8 (21.1%) | 10 (27.8%) |  |  |
| **Intervention** |  |  | 0.4 | 0.079 |
| Non-pharmacological | 32 (84.2%) | 24 (66.7%) |  |  |
| Pharmacological | 6 (15.8%) | 12 (33.3%) |  |  |
| **Primary purpose** |  |  | 0.9 | 0.008 |
| Treatment | 32 (84.2%) | 20 (55.6%) |  |  |
| Prevent | 2 (5.3%) | 7 (19.4%) |  |  |
| Support care | 3 (7.9%) | 1 (2.8%) |  |  |
| Other | 1 (2.6%) | 8 (22.2%) |  |  |
| **Study design** |  |  | 0.7 | 0.025 |
| Parallel group | 36 (94.7%) | 28 (77.8%) |  |  |
| Crossover | 1 (2.6%) | 8 (22.2%) |  |  |
| Factorial | 1 (2.6%) | 0 (0.0%) |  |  |
| **No. of arms** |  |  | 0 | 0.863 |
| 2 | 30 (78.9%) | 29 (80.6%) |  |  |
| ≥3 | 8 (21.1%) | 7 (19.4%) |  |  |
| **Blinding** |  |  | 0.5 | 0.123 |
| None/open label | 20 (52.6%) | 20 (55.6%) |  |  |
| Single | 5 (13.2%) | 10 (27.8%) |  |  |
| Double or more | 13 (34.2%) | 6 (16.7%) |  |  |
| **Economic of PI region** |  |  | 0.2 | 0.418 |
| LMIC | 8 (21.1%) | 5 (13.9%) |  |  |
| HIC | 30 (78.9%) | 31 (86.1%) |  |  |
| **Recruitment region** |  |  | 0.3 | 0.246 |
| Non-Asian | 30 (78.9%) | 32 (88.9%) |  |  |
| Asian | 8 (21.1%) | 4 (11.1%) |  |  |
| **Recruitment region** |  |  | 0.4 | 0.732 |
| Africa | 1 (2.6%) | 2 (5.6%) |  |  |
| Asian | 8 (21.1%) | 4 (11.1%) |  |  |
| European | 19 (50.0%) | 21 (58.3%) |  |  |
| North America | 6 (15.8%) | 7 (19.4%) |  |  |
| South America | 1 (2.6%) | 1 (2.8%) |  |  |
| Oceanian | 3 (7.9%) | 1 (2.8%) |  |  |
| **Operation** |  |  | 0.1 | 0.894 |
| Sleeve Gastrectomy | 10 (26.3%) | 11 (30.6%) |  |  |
| Roux-en-Y-bypass | 21 (55.3%) | 18 (50.0%) |  |  |
| Both | 7 (18.4%) | 7 (19.4%) |  |  |
| **Funding type** |  |  | 0.2 | 0.377 |
| None/departmental | 31 (81.6%) | 32 (88.9%) |  |  |
| Industry/other | 7 (18.4%) | 4 (11.1%) |  |  |
| **No. of participants** |  |  | 0.3 | 0.159 |
| ≤100 | 17 (44.7%) | 22 (61.1%) |  |  |
| ＞100 | 21 (55.3%) | 14 (38.9%) |  |  |
| **No. of participants** |  |  | 0 | 0.843 |
| ≤200 | 31 (81.6%) | 30 (83.3%) |  |  |
| ＞200 | 7 (18.4%) | 6 (16.7%) |  |  |

**Table S6. Characteristics of Randomized Clinical Trials according to presence of guideline citation**

|  | **Absence of citing by guidelines（N=48）** | **Presence of citing by guidelines（N=26）** | **SMD** | **P-value** |
| --- | --- | --- | --- | --- |
| **Enrollment Time** | 37.8 ± 37.7 | 50.6 ± 43.8 | 0.3 | 0.193 |
| **Enrollment Time** |  |  | 0.9 | <0.001 |
| 2000-2010 | 4 (8.3%) | 12 (46.2%) |  |  |
| After2010 | 44 (91.7%) | 14 (53.8%) |  |  |
| **No. of centres** |  |  | 0.4 | 0.064 |
| Monocentric | 39 (81.2%) | 16 (61.5%) |  |  |
| Multicenter | 9 (18.8%) | 10 (38.5%) |  |  |
| **Primary Outcome measures** |  |  | 0.2 | 0.341 |
| Non-weight loss | 33 (68.8%) | 15 (57.7%) |  |  |
| Weight loss | 15 (31.2%) | 11 (42.3%) |  |  |
| **Primary Outcome measures** |  |  | 0.4 | 0.143 |
| Non-anesthesia | 38 (79.2%) | 24 (92.3%) |  |  |
| Anesthesia | 10 (20.8%) | 2 (7.7%) |  |  |
| **Primary Outcome measures** |  |  | 0.4 | 0.129 |
| Non-comorbility | 39 (81.2%) | 17 (65.4%) |  |  |
| Comorbility | 9 (18.8%) | 9 (34.6%) |  |  |
| **Intervention** |  |  | 0.2 | 0.452 |
| Non-pharmacological | 35 (72.9%) | 21 (80.8%) |  |  |
| Pharmacological | 13 (27.1%) | 5 (19.2%) |  |  |
| **Primary purpose** |  |  | 0.5 | 0.232 |
| Treatment | 30 (62.5%) | 22 (84.6%) |  |  |
| Prevent | 7 (14.6%) | 2 (7.7%) |  |  |
| Support care | 3 (6.2%) | 1 (3.8%) |  |  |
| Other | 8 (16.7%) | 1 (3.8%) |  |  |
| **Study design** |  |  | 0.3 | 0.392 |
| Parallel group | 42 (87.5%) | 22 (84.6%) |  |  |
| Crossover | 6 (12.5%) | 3 (11.5%) |  |  |
| Factorial | 0 (0.0%) | 1 (3.8%) |  |  |
| **No. of arms** |  |  | 0.2 | 0.442 |
| 2 | 37 (77.1%) | 22 (84.6%) |  |  |
| ≥3 | 11 (22.9%) | 4 (15.4%) |  |  |
| **Blinding** |  |  | 0.4 | 0.378 |
| None/open label | 24 (50.0%) | 16 (61.5%) |  |  |
| Single | 12 (25.0%) | 3 (11.5%) |  |  |
| Double or more | 12 (25.0%) | 7 (26.9%) |  |  |
| **Economic of PI region** |  |  | 0.1 | 0.782 |
| LMIC | 8 (16.7%) | 5 (19.2%) |  |  |
| HIC | 40 (83.3%) | 21 (80.8%) |  |  |
| **Recruitment region** |  |  | 0.2 | 0.422 |
| Non-Asian | 39 (81.2%) | 23 (88.5%) |  |  |
| Asian | 9 (18.8%) | 3 (11.5%) |  |  |
| **Recruitment region** |  |  | 0.4 | 0.77 |
| Africa | 1 (2.1%) | 2 (7.7%) |  |  |
| Asian | 9 (18.8%) | 3 (11.5%) |  |  |
| European | 26 (54.2%) | 14 (53.8%) |  |  |
| North America | 9 (18.8%) | 4 (15.4%) |  |  |
| South America | 1 (2.1%) | 1 (3.8%) |  |  |
| Oceanian | 2 (4.2%) | 2 (7.7%) |  |  |
| **Operation** |  |  | 0.3 | 0.405 |
| Sleeve Gastrectomy | 15 (31.2%) | 6 (23.1%) |  |  |
| Roux-en-Y-bypass | 26 (54.2%) | 13 (50.0%) |  |  |
| Both | 7 (14.6%) | 7 (26.9%) |  |  |
| **Funding type** |  |  | 0 | 0.926 |
| None/departmental | 41 (85.4%) | 22 (84.6%) |  |  |
| Industry/other | 7 (14.6%) | 4 (15.4%) |  |  |
| **No. of participants** |  |  | 0.2 | 0.527 |
| ≤100 | 24 (50.0%) | 15 (57.7%) |  |  |
| ＞100 | 24 (50.0%) | 11 (42.3%) |  |  |
| **No. of participants** |  |  | 0.4 | 0.1 |
| ≤200 | 37 (77.1%) | 24 (92.3%) |  |  |
| ＞200 | 11 (22.9%) | 2 (7.7%) |  |  |
| **Impact Factor** |  |  | 0.8 | 0.001 |
| ≤10 | 41 (85.4%) | 13 (50.0%) |  |  |
| ＞10 | 7 (14.6%) | 13 (50.0%) |  |  |
| **Results as assumed*** |  |  | 0.3 | 0.266 |
| Absence | 15 (31.2%) | 5 (19.2%) |  |  |
| Presence | 33 (68.8%) | 21 (80.8%) |  |  |
| **Research waste** |  |  | 0.8 | 0.002 |
| Absence | 12 (25.0%) | 16 (61.5%) |  |  |
| Presence | 36 (75.0%) | 10 (38.5%) |  |  |

**Table S7. Adjusted logistic regression analysis of association of key study characteristics with presence of guideline citation**

|  | **Univariate analysis** | | | **Multivariate analysis** | | |
| --- | --- | --- | --- | --- | --- | --- |
|  | OR | 95%CI | **P** | OR | 95%CI | **P** |
| **Enrollment Time** | 1.008 | 0.996-1.02 | 0.198 |  |  |  |
| **Registration time** |  |  |  |  |  |  |
| 2000-2010 |  | ref |  |  |  |  |
| After2010 | 0.106 | 0.029-0.382 | 0.001 | 0.063 | 0.014-0.283 | 0.001 |
| **No. of centres** |  |  |  |  |  |  |
| Monocentric |  | ref |  |  |  |  |
| Multicenter | 2.708 | 0.927-7.913 | 0.069 |  |  |  |
| **Primary Outcome measures** |  |  |  |  |  |  |
| Non-weight loss |  | ref |  |  |  |  |
| Weight loss | 1.613 | 0.6-4.337 | 0.343 |  |  |  |
| **Primary Outcome measures** |  |  |  |  |  |  |
| Non-anesthesia |  | ref |  |  |  |  |
| Anesthesia | 0.317 | 0.064-1.571 | 0.159 |  |  |  |
| **Primary Outcome measures** |  |  |  |  |  |  |
| Non-comorbility |  | ref |  |  |  |  |
| Comorbility | 2.294 | 0.775-6.792 | 0.134 |  |  |  |
| **Intervention** |  |  |  |  |  |  |
| Non-pharmacological |  | ref |  |  |  |  |
| Pharmacological | 0.641 | 0.2-2.054 | 0.454 |  |  |  |
| **Primary purpose** |  |  |  |  |  |  |
| Non-Treatment |  | ref |  |  |  |  |
| Treatment | 3.3 | 0.979-11.123 | 0.054 |  |  |  |
| **Study design** |  |  |  |  |  |  |
| Parallel group |  |  |  |  |  |  |
| Non-parallel group | 1.273 | 0.325-4.99 | 0.729 |  |  |  |
| **No. of arms** |  |  |  |  |  |  |
| 2 |  | ref |  |  |  |  |
| ≥3 | 0.612 | 0.173-2.156 | 0.444 |  |  |  |
| **Blinding** |  |  | 0.395 |  |  |  |
| None/open label |  | ref |  |  |  |  |
| Single | 0.375 | 0.091-1.543 | 0.174 |  |  |  |
| Double or more | 0.875 | 0.284-2.699 | 0.816 |  |  |  |
| **Economic of PI region** |  |  |  |  |  |  |
| LMIC |  | ref |  |  |  |  |
| HIC | 0.84 | 0.244-2.891 | 0.782 |  |  |  |
| **Recruitment region** |  |  |  |  |  |  |
| Non-Asian |  |  |  |  |  |  |
| Asian | 0.565 | 0.139-2.303 | 0.426 |  |  |  |
| **Operation** |  |  | 0.414 |  |  |  |
| SleeveGastrectomy |  | ref |  |  |  |  |
| Roux-en-Y-bypass | 1.25 | 0.393-3.977 | 0.706 |  |  |  |
| Both | 2.5 | 0.609-10.261 | 0.203 |  |  |  |
| **Funding type** |  |  |  |  |  |  |
| None/departmental |  | ref |  |  |  |  |
| Industry/other | 1.065 | 0.281-4.04 | 0.926 |  |  |  |
| **No. of participants** |  |  |  |  |  |  |
| ＜100 |  | ref |  |  |  |  |
| ≥100 | 0.733 | 0.28-1.919 | 0.527 |  |  |  |
| **No. of participants** |  |  |  |  |  |  |
| ＜200 |  | ref |  |  |  |  |
| ≥200 | 0.28 | 0.057-1.377 | 0.117 |  |  |  |
| **Results as assumed*** |  |  |  |  |  |  |
| Absence |  | ref |  |  |  |  |
| Presence | 1.909 | 0.604-6.032 | 0.271 |  |  |  |
| **Research waste** |  |  |  |  |  |  |
| Absence |  | ref |  |  |  |  |
| Presence | 0.208 | 0.075-0.581 | 0.003 | 0.124 | 0.035-0.446 | 0.001 |

*The primary endpoints reported were as assumed.

**Table S8 .Characteristics of Randomized Clinical Trials according to reuse of prospective data**

|  | **Absence of reuse of prospective data（N=55）** | **Presence of reuse of prospective data（N=19）** | **SMD** | **P-value** |
| --- | --- | --- | --- | --- |
| **Enrollment Time** | 31.4 ± 29.4 | 73.9 ± 50.1 | 1 | <0.001 |
| **Registration time** |  |  | 0.6 | 0.012 |
| 2000-2010 | 8 (14.5%) | 8 (42.1%) |  |  |
| After2010 | 47 (85.5%) | 11 (57.9%) |  |  |
| **No. of centres** |  |  | 0.5 | 0.057 |
| Monocentric | 44 (80.0%) | 11 (57.9%) |  |  |
| Multicenter | 11 (20.0%) | 8 (42.1%) |  |  |
| **Primary Outcome measures** |  |  | 0.7 | 0.016 |
| Non-weight loss | 40 (72.7%) | 8 (42.1%) |  |  |
| Weight loss | 15 (27.3%) | 11 (57.9%) |  |  |
| **Primary Outcome measures** |  |  | 0.7 | 0.026 |
| Non-anesthesia | 43 (78.2%) | 19 (100.0%) |  |  |
| Anesthesia | 12 (21.8%) | 0 (0.0%) |  |  |
| **Primary Outcome measures** |  |  | 0.4 | 0.14 |
| Non-comorbility | 44 (80.0%) | 12 (63.2%) |  |  |
| Comorbility | 11 (20.0%) | 7 (36.8%) |  |  |
| **Intervention** |  |  | 0.7 | 0.025 |
| Non-pharmacological | 38 (69.1%) | 18 (94.7%) |  |  |
| Pharmacological | 17 (30.9%) | 1 (5.3%) |  |  |
| **Primary purpose** |  |  | 0.3 | 0.722 |
| Treatment | 37 (67.3%) | 15 (78.9%) |  |  |
| Prevent | 7 (12.7%) | 2 (10.5%) |  |  |
| Support care | 3 (5.5%) | 1 (5.3%) |  |  |
| Other | 8 (14.5%) | 1 (5.3%) |  |  |
| **Study design** |  |  | 0.4 | 0.461 |
| Parallel group | 46 (83.6%) | 18 (94.7%) |  |  |
| Crossover | 8 (14.5%) | 1 (5.3%) |  |  |
| Factorial | 1 (1.8%) | 0 (0.0%) |  |  |
| **No. of arms** |  |  | 0 | 0.922 |
| 2 | 44 (80.0%) | 15 (78.9%) |  |  |
| ≥3 | 11 (20.0%) | 4 (21.1%) |  |  |
| **Blinding** |  |  | 0.2 | 0.849 |
| None/open label | 29 (52.7%) | 11 (57.9%) |  |  |
| Single | 12 (21.8%) | 3 (15.8%) |  |  |
| Double or more | 14 (25.5%) | 5 (26.3%) |  |  |
| **Economic of PI region** |  |  | 0.1 | 0.643 |
| LMIC | 9 (16.4%) | 4 (21.1%) |  |  |
| HIC | 46 (83.6%) | 15 (78.9%) |  |  |
| **Recruitment region** |  |  | 0 | 0.953 |
| Non-Asian | 46 (83.6%) | 16 (84.2%) |  |  |
| Asian | 9 (16.4%) | 3 (15.8%) |  |  |
| **Recruitment region** |  |  | 0.6 | 0.205 |
| Africa | 3 (5.5%) | 0 (0.0%) |  |  |
| Asian | 9 (16.4%) | 3 (15.8%) |  |  |
| European | 31 (56.4%) | 9 (47.4%) |  |  |
| North America | 9 (16.4%) | 4 (21.1%) |  |  |
| South America | 0 (0.0%) | 2 (10.5%) |  |  |
| Oceanian | 3 (5.5%) | 1 (5.3%) |  |  |
| **Operation** |  |  | 0.5 | 0.252 |
| Sleeve Gastrectomy | 18 (32.7%) | 3 (15.8%) |  |  |
| Roux-en-Y-bypass | 26 (47.3%) | 13 (68.4%) |  |  |
| Both | 11 (20.0%) | 3 (15.8%) |  |  |
| **Funding type** |  |  | 0.6 | 0.018 |
| None/departmental | 50 (90.9%) | 13 (68.4%) |  |  |
| Industry/other | 5 (9.1%) | 6 (31.6%) |  |  |
| **No. of participants** |  |  | 0.1 | 0.599 |
| ≤100 | 28 (50.9%) | 11 (57.9%) |  |  |
| ＞100 | 27 (49.1%) | 8 (42.1%) |  |  |
| **No. of participants** |  |  | 0.1 | 0.813 |
| ≤200 | 45 (81.8%) | 16 (84.2%) |  |  |
| ＞200 | 10 (18.2%) | 3 (15.8%) |  |  |
| **Impact Factor** |  |  | 1.4 | <0.001 |
| ＜10 | 48 (87.3%) | 6 (31.6%) |  |  |
| ≥10 | 7 (12.7%) | 13 (68.4%) |  |  |
| **Results as assumed*** |  |  | 0.6 | 0.06 |
| Absence | 18 (32.7%) | 2 (10.5%) |  |  |
| Presence | 37 (67.3%) | 17 (89.5%) |  |  |
| **Research waste** |  |  | 0.6 | 0.037 |
| Absence | 17 (30.9%) | 11 (57.9%) |  |  |
| Presence | 38 (69.1%) | 8 (42.1%) |  |  |
