# Supplementary material for: Characterization changes and research waste in randomized controlled trials of global bariatric surgery over the past 20 years: cross-sectional study
Source: Int J Surg. 2023 Dec 18;110(3):1420–9. doi: 10.1097/JS9.0000000000001013 (PMC10942146; doi:10.1097/JS9.0000000000001013)
Supplement: SUPPLEMENTARY MATERIAL [file js9-110-1420-s006.docx]

**Table S9.****Two step adjusted logistic regression analysis of the effect of key study characteristics on presence of reuse of prospective data.**

|  | **Univariate analysis** | | **Multivariate analysis first step** | | **Multivariate analysis second step** | |
| --- | --- | --- | --- | --- | --- | --- |
|  | OR（95% CI) | **P** | OR（95% CI) | **P** | OR（95% CI) | **P** |
| **Enrollment Time** | 1.027(1.011-1.044) | 0.001 | 1.027（1.011-1.044） | 0.001 |  |  |
| **Registration time** |  |  |  |  |  |  |
| 2000-2010 |  |  |  |  |  |  |
| After2010 | 0.234(0.072-0.761) | 0.016 |  |  | 0.205（0.059-0.718） | 0.013 |
| **No. of centres** |  |  |  |  |  |  |
| Monocentric |  |  |  |  |  |  |
| Multicenter | 2.909(0.944-8.962) | 0.063 |  |  |  |  |
| **Primary Outcome measures** |  |  |  |  |  |  |
| Non-weight loss |  |  |  |  |  |  |
| Weight loss | 3.667(1.237-10.873) | 0.019 |  |  |  |  |
| **Primary Outcome measures** |  |  |  |  |  |  |
| Non-comorbility |  |  |  |  |  |  |
| Comorbility | 2.333(0.744-7.314) | 0.146 |  |  |  |  |
| **Intervention** |  |  |  |  |  |  |
| Non-pharmacological |  |  |  |  |  |  |
| Pharmacological | 0.223(0.047-1.068) | 0.06 |  |  |  |  |
| **Primary purpose** |  |  |  |  |  |  |
| Non-pharmacological |  |  |  |  |  |  |
| Pharmacological | 1.824(0.529-6.294) | 0.341 |  |  |  |  |
| **Study design** |  |  |  |  |  |  |
| Parallel group |  |  |  |  |  |  |
| Non-parallel group | 0.284(0.034-2.405) | 0.248 |  |  |  |  |
| **No. of arms** |  |  |  |  |  |  |
| 2 |  |  |  |  |  |  |
| ≥3 | 1.067(0.295-3.858) | 0.922 |  |  |  |  |
| **Blinding** |  | 0.85 |  |  |  |  |
| None/open label |  |  |  |  |  |  |
| Single | 0.659(0.156-2.79) | 0.571 |  |  |  |  |
| Double or more | 0.942(0.274-3.236) | 0.924 |  |  |  |  |
| **Economic of PI region** |  |  |  |  |  |  |
| LMIC |  |  |  |  |  |  |
| HIC | 0.734(0.197-2.73) | 0.644 |  |  |  |  |
| **Recruitment region** |  |  |  |  |  |  |
| Non-Asian |  |  |  |  |  |  |
| Asian | 0.958(0.23-3.985) | 0.953 |  |  |  |  |
| **Operation** |  | 0.267 |  |  |  |  |
| SleeveGastrectomy |  |  |  |  |  |  |
| Roux-en-Y-bypass | 3(0.746-12.066) | 0.122 |  |  |  |  |
| Both | 1.636(0.279-9.582) | 0.585 |  |  |  |  |
| **Funding type** |  |  |  |  |  |  |
| None/departmental |  |  |  |  |  |  |
| Industry/other | 4.615(1.215-17.529) | 0.025 |  |  |  |  |
| **No. of participants** |  |  |  |  |  |  |
| ＜100 |  |  |  |  |  |  |
| ≥100 | 1.146(0.399-3.288) | 0.8 |  |  |  |  |
| **No. of participants** |  |  |  |  |  |  |
| ＜200 |  |  |  |  |  |  |
| ≥200 | 0.606(0.152-2.411) | 0.477 |  |  |  |  |
| **Results as assumed*** |  |  |  |  |  |  |
| Absence |  |  |  |  |  |  |
| Presence | 4.135(0.861-19.87) | 0.076 |  |  |  |  |
| **Research waste** |  |  |  |  |  |  |
| Absence |  |  |  |  |  |  |
| Presence | 0.325(0.111-0.954) | 0.041 |  |  | 0.286（0.090-0.903） | 0.033 |
